# Successful laparoscopic arterial ligation of splenic artery aneurysm with a splenomesenteric trunk: a case report and literature review

**DOI:** 10.1186/s40792-024-02051-0

**Published:** 2024-11-01

**Authors:** Shigeya Takeo, Hideki Izumi, Hisamichi Yoshii, Rika Fjino, Masaya Mukai, Hidekazu Furuya, Akiyoshi Yamamoto, Shunsuke Kamei, Yukihisa Ogawa, Terumitsu Hasebe, Junichi Kaneko, Hiroyasu Makuuchi

**Affiliations:** 1 Department of Gastrointestinal Surgery, Tokai University Hachioji Hospital, 1838 Ishikawa, Hachioji, Tokyo 192-0032 Japan; 2 Department of Cardiovascular Surgery, Tokai University Hachioji Hospital, 1838 Ishikawa, Hachioji, Tokyo 192-0032 Japan; 3 Department of Radiology, Tokai University Hachioji Hospital, 1838 Ishikawa, Hachioji, Tokyo 192-0032 Japan

**Keywords:** Splenic artery aneurysm, Splenomesenteric trunk, Laparoscopic arterial ligation

## Abstract

**Background:**

The mortality rate of splenic artery aneurysm rupture is very high, and patients with aneurysms larger than 30 mm are recommended for treatment, regardless of the presence or absence of symptoms. We herein report a case of splenic artery aneurysm with an abnormal bifurcation that was treated with laparoscopic ligation of the splenic artery.

**Case presentation:**

A 51 year-old Japanese male was referred to our hospital because a splenic artery aneurysm was noted on abdominal echocardiography during a medical examination. The splenic artery bifurcated from the superior mesenteric artery (SMA), and a 38-mm splenic artery aneurysm was found just after the bifurcation; thus, surgery was performed. Intraoperative angiography was performed, a balloon catheter was placed before the splenic artery bifurcation, and laparoscopic splenic artery ligation was performed to prepare for sudden bleeding. After ligation of the splenic artery, angiography was performed again to confirm the absence of the splenic artery aneurysm and that the peripheral splenic artery was visible through the peripheral collateral vessels. The patient was discharged on the fourth postoperative day, with good progress. Contrast-enhanced computed tomography performed 1 month postoperatively confirmed the disappearance of the splenic artery aneurysm, and the contrast-enhanced peripheral splenic artery was visible.

**Conclusion:**

This is the first report of a safe laparoscopic artery ligation procedure for a splenic artery aneurysm with an abnormal splenic artery bifurcation from the SMA, in which a balloon catheter was placed at the splenic artery bifurcation.

## Background

The splenic, common hepatic, and left gastric arteries branch from the celiac artery, and 84% of these branches originate from the celiac artery. Meanwhile, splenic artery bifurcations from the superior mesenteric artery (SMA) account for approximately 1% of all splenic artery bifurcations [1]. A major characteristic of splenic artery aneurysms with an abnormal bifurcation is that all aneurysms are located immediately after the bifurcation [2]. The mortality rate is as high as 30% when a splenic artery aneurysm ruptures, even with therapeutic intervention; thus, appropriate therapeutic intervention is considered necessary [3]. According to the Society for Vascular Surgery guidelines, treatment is required for pseudoaneurysms larger than 30 mm, whether symptomatic or asymptomatic [4]. Herein, we report a case of splenic artery aneurysm with an abnormal bifurcation that was treated with laparoscopic ligation of the splenic artery.

## Case presentation

The patient is a 51 year-old Japanese male who was referred to our hospital for cardiovascular surgery approximately 10 years ago because of a splenic artery aneurysm noted on abdominal echocardiography during a medical examination. The splenic aneurysm was 32 mm in size when he visited our hospital; thus, we recommended treatment. However, the patient was against it, and thus, we monitored his condition through periodic follow-up. The splenic artery aneurysm had gradually increased in size and had grown to 38 mm; thus, the patient decided to undergo treatment.

His medical history included hyperuricemia, but there were no other relevant medical conditions or trauma. The blood biochemical findings on admission were within normal limits.

**Fig. 1:**
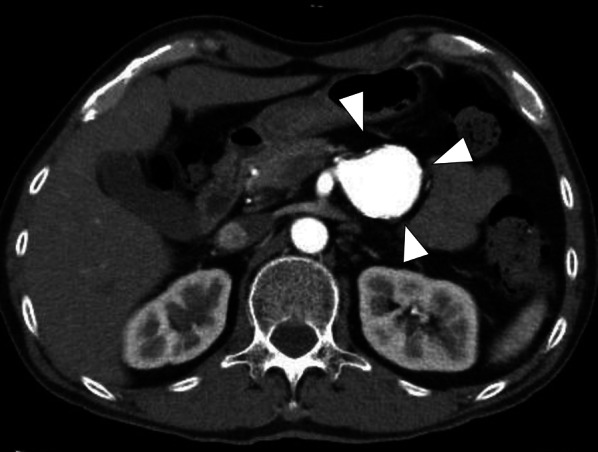
Arterial-phase computed tomography scan showing a homogeneously contrasted aneurysm (white triangle)

**Fig. 2:**
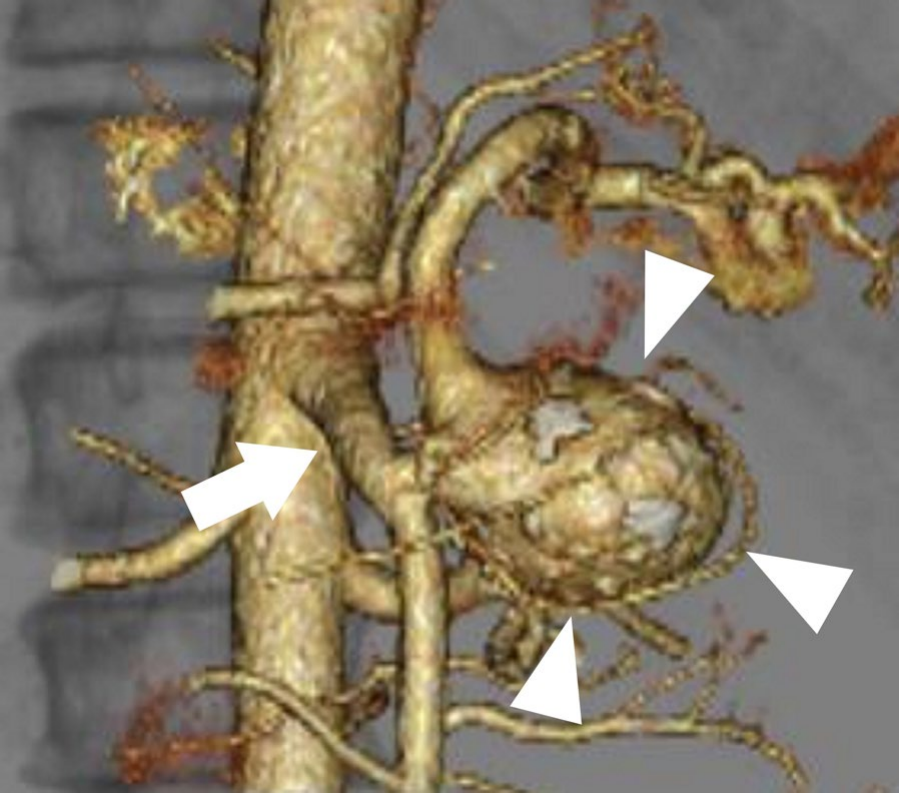
Preoperative computed tomographic angiography findings. The splenic artery bifurcated from the superior mesenteric artery (white arrow), and a 38-mm splenic artery aneurysm (white triangle) was visible immediately after the bifurcation

Arterial-phase computed tomography (CT) revealed a homogeneously stained splenic artery aneurysm (Fig. 1). Three-dimensional CT revealed that the splenic artery bifurcated from the SMA, and a 38-mm large aneurysm was observed immediately after the bifurcation (Fig. 2).

**Fig. 3:**
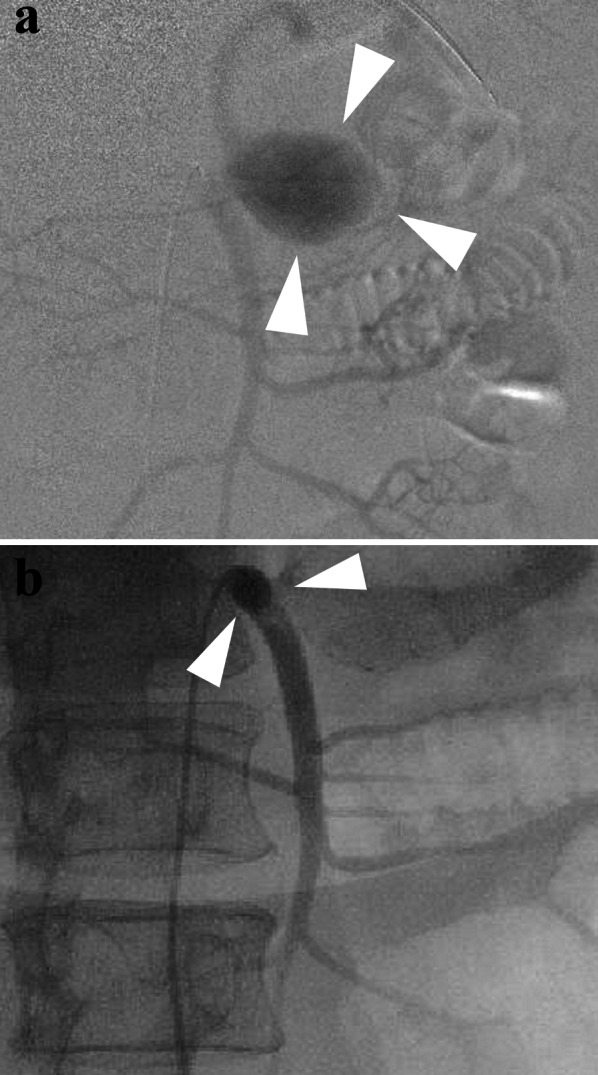
Angiography during laparoscopic surgery. **a** Intraoperative angiography showing an aneurysm (white triangle) in the splenic artery branching from the superior mesenteric artery. **b** A balloon catheter (white triangle) was placed immediately before the bifurcation of the splenic artery aneurysm to control bleeding

Under general anesthesia, a catheter was inserted through the right femoral artery to identify the splenic artery branching from the SMA and the splenic artery aneurysm (Fig. 3a). To prepare for the rupture of the splenic artery aneurysm by laparoscopic manipulation, a deflated balloon catheter (5.2 Fr Selecon MP Catheter II with a balloon diameter of 20 mm) was placed at the origin of the SMA to allow for hemostasis in case of sudden bleeding (Fig. 3b). There was no heparin used in this case for thromboprophylaxis.

**Fig. 4:**
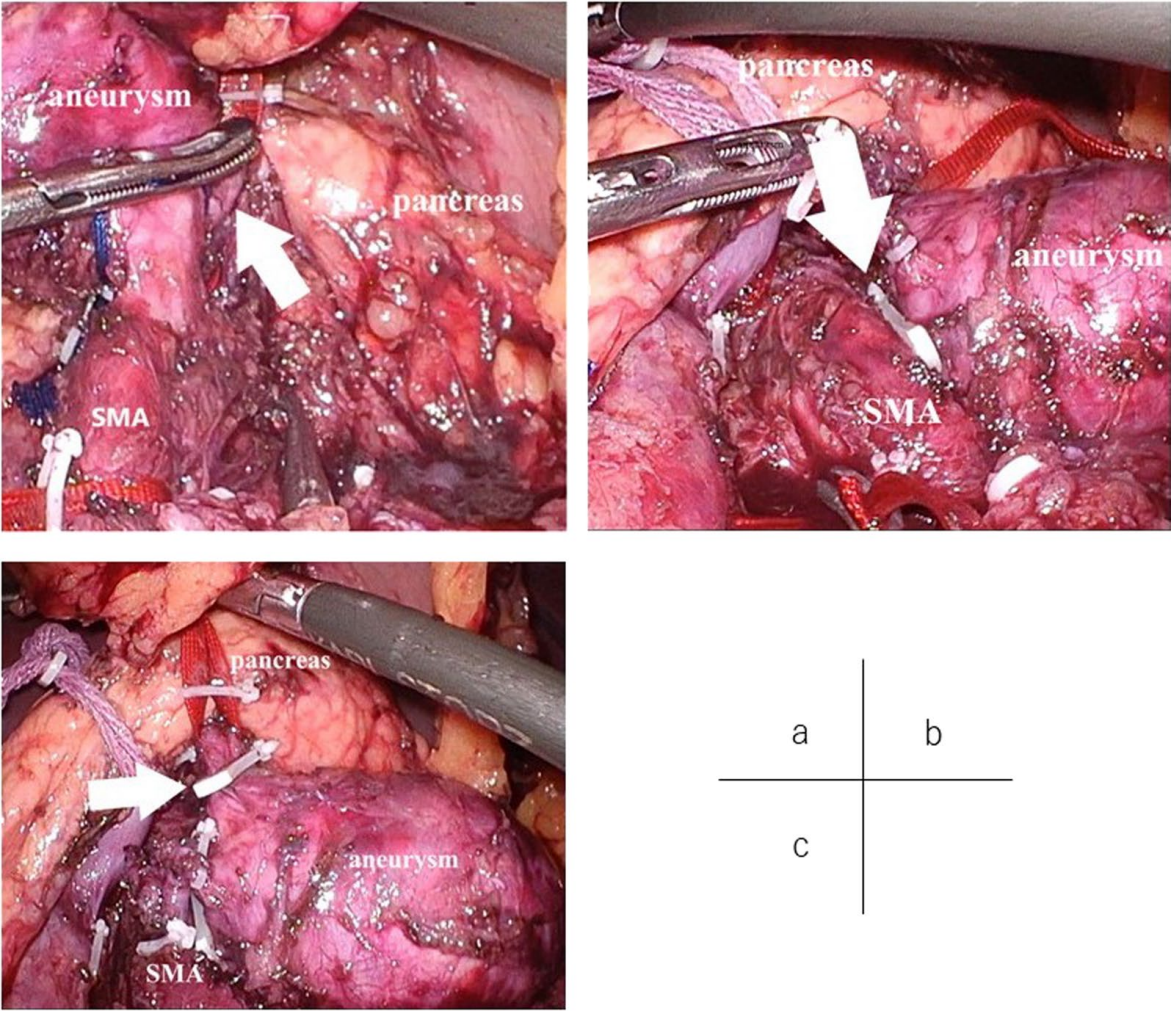
Findings during laparoscopic surgery. **a** The splenic artery was taped to the distal side of the aneurysm. **b** The splenic artery was ligated on the proximal side. **c** The splenic artery was ligated on the distal sides of the splenic artery aneurysm

**Fig. 5:**
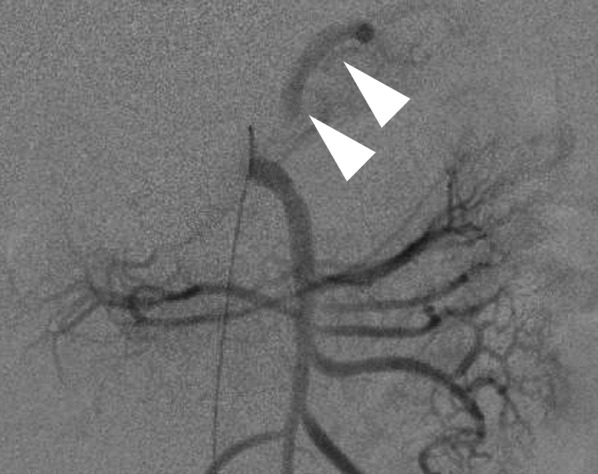
Postoperative angiography. No splenic artery aneurysm was visualized, but the distal splenic artery was contrasted

The gastrojejunal mesentery was opened, the stomach was lifted, and the pancreas was identified. The pancreas was tunneled immediately above the superior mesenteric vein, and the pancreas and splenic vein were taped together. The aneurysm was identified, and the splenic artery was taped to the distal side of the aneurysm (Fig. 4a). After taping the main trunk of the SMA, the splenic artery was taped to the proximal side of the aneurysm. The Hem-o-lok XL (Teleflex, NC, USA) was used to ligate the splenic artery on the proximal (Fig. 4b) and distal sides of the splenic artery aneurysm (Fig. 4c). Angiography was performed to confirm that no splenic artery or aneurysm was visible through the main trunk of the SMA and that the distal splenic artery and spleen were visible (Fig. 5). The operation time was 240 min, and blood loss was 30 mL.

**Fig. 6:**
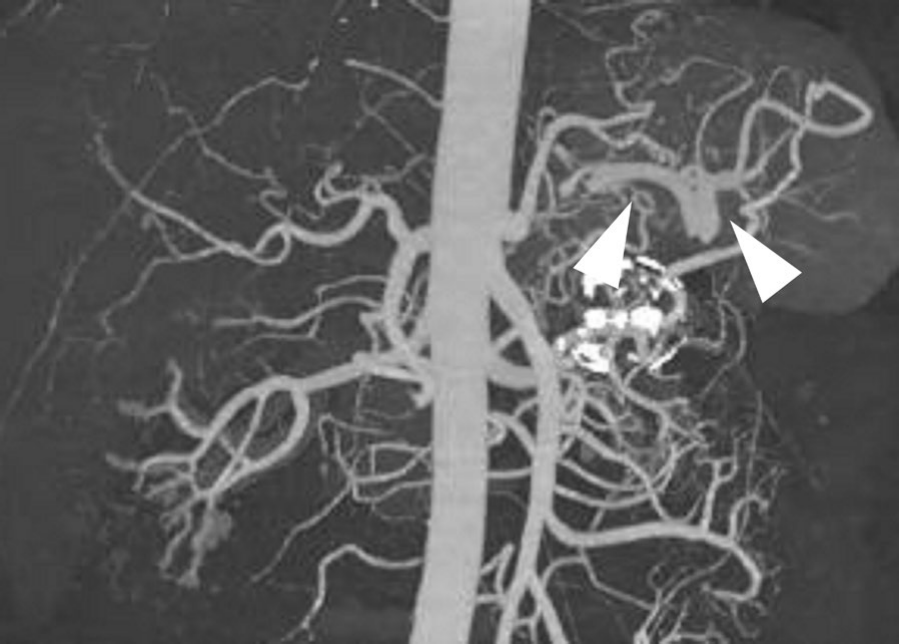
Contrast-enhanced computed tomography scan at 1 month postoperatively. The splenic artery aneurysm completely disappeared, and the distal splenic artery (white triangle) was visible

The patient was discharged on the fourth postoperative day, with good progress. Contrast-enhanced CT scan performed 1 month postoperatively confirmed the disappearance of the splenic artery aneurysm, and the distal splenic artery was visible (Fig. 6).

## Discussion

The prevalence rate of abdominal visceral aneurysms is approximately 0.1–2% [3], and splenic artery aneurysms are the most common abdominal visceral aneurysms, accounting for 60% of all cases [5]. Although other abdominal visceral aneurysms are more common in males, splenic artery aneurysms are more common in females (1:4 ratio). Estrogen and other female hormones may be contributing factors [6].

Ekingen et al. [6] classified splenic artery bifurcations into 22 types using CT angiography, and splenic artery bifurcation from the SMA is classified as type 8 and is present in 0.13% of 750 patients. Aneurysm formation in the splenic artery with abnormal bifurcation, as in the present case, is extremely rare, with fewer than 50 cases reported so far [7, 8]. Splenic artery aneurysms without branching abnormalities are more common on the distal side [9–11]. In contrast, splenic artery aneurysms with an abnormal bifurcation are characterized by the fact that all aneurysms reported to date are located at the origin [2]. Splenic artery aneurysms are often associated with atherosclerosis, pancreatitis, essential and portal hypertension, pregnancy, and other conditions in the literature. They are often found incidentally, and most are asymptomatic [12]. However, the patient in our case was male and had no history of aneurysms that could have caused them, as described above. The abnormal splenic artery bifurcated proximal to the SMA and rose at a right angle. It has been hypothesized that congenital alterations, such as arterial media dysplasia and sudden hemodynamic changes resulting from this anomaly, could be the primary causes of splenic artery abnormalities with this anatomic anomaly [2, 12].

Several studies have reported the treatment for splenic artery aneurysms branching from the SMA, including open aneurysm ligation and resection [13, 14], and aneurysm resection with revascularization and splenectomy [15], as well as coil embolization [7] and stent graft insertion [2], which have recently been reported with the development of endovascular treatment techniques. Hogendoorn et al. [16] reported that endovascular treatment has better short-term results and lower perioperative mortality rates than open surgery, but it also has a high reintervention rate due to late complications. Specific complications of endovascular treatment include coil migration, splenic infarction, post-embolization syndrome, and revascularization [16, 17]. In splenic artery aneurysms with abnormal bifurcation, the indication for endovascular treatment should be carefully considered, because if the coil moves into the SMA, there may be intestinal necrosis [8]. In recent years, the placement of a Viabahn stent graft in the main trunk of the SMA to close an aneurysm has been reported [18]. However, stent grafts alone do not completely stop blood flow into the aneurysm from the periphery, and this method may be inadequate. Kuwada et al. [19] reported stent grafting of the SMA and peripheral splenic artery coiling. We have also reported the usefulness of the Viabahn stent graft for bleeding from gastroduodenal artery (GDA) pseudoaneurysms after pancreaticoduodenectomy [20]; even if a stent graft placed in the common hepatic artery becomes occluded, the liver is highly resistant to ischemia and can tolerate it until collateral blood vessels develop. Thus, the Viabahn stent graft for pseudoaneurysms from the GDA is useful for achieving hemostasis and saving lives. However, stent occlusion in SMA main stem stent grafting is likely to cause intestinal necrosis and other problems and should be avoided if possible.

## Conclusion

Kakamad et al. [8] reported a case of a splenic artery aneurysm with an abnormal bifurcation that was treated with open ligation. In the present case, the same procedure was performed laparoscopically. A balloon catheter was placed in the main trunk of the SMA during the laparoscopic procedure to prepare for sudden bleeding in case of aneurysm rupture during laparoscopic manipulation. To our knowledge, this is the first report of a safe laparoscopic artery ligation procedure for a splenic artery aneurysm branching from the SMA.

## Data Availability

Not applicable.

## Abbreviations

CT: Computed tomography
SMA: Superior mesenteric artery
GDA: Gastroduodenal artery

## References

[1] Lippert H, Pabst R. Arterial variations in man: classification and frequency. München: JF Bergmann Verlag; 1985. p. 30–41.

[2] Liu Q, Lu JP, Wang F, Wang L, Jin AG, Wang J, et al. Detection of anomalous splenic artery aneurysms with three-dimensional contrast-enhanced MR angiography. Abdom Imag. 2009;34:772–6. 10.1007/s00261-008-9467-6. 10.1007/s00261-008-9467-6 18949507

[3] Pitton MB, Dappa E, Jungmann F, Kloeckner R, Schotten S, Wirth GM, et al. Visceral artery aneurysms: incidence, management, and outcome analysis in a tertiary care center over one decade. Eur Radiol. 2015;25:2004–14. 10.1007/s00330-015-3599-1. 25693662 10.1007/s00330-015-3599-1 PMC4457909

[4] Chaer RA, Abularrage CJ, Coleman DM, Eslami MH, Kashyap VS, Rockman C, et al. The society for vascular surgery clinical practice guidelines on the management of visceral aneurysms. J Vasc Surg. 2020;72:3S-39S. 10.1016/j.jvs.2020.01.039. 32201007 10.1016/j.jvs.2020.01.039

[5] Stanley JC, Wakefield TW, Graham LM, Whitehouse WM Jr, Zelenock GB, Lindenauer SM. Clinical importance and management of splanchnic artery aneurysms. J Vasc Surg. 1986;3:836–40. 3701947

[6] Ekingen A, Hatipoğlu ES, Hamidi C, Tuncer MC, Ertuğrul Ö. Splenic artery angiography: clinical classification of origin and branching variations of splenic artery by multi-detector computed tomography angiography method. Folia Morphol. 2020;79:236–46. 10.5603/FM.a2019.0088. 10.5603/FM.a2019.0088 31436304

[7] Ichikawa Y, Hosoi Y, Ikezoe T, Isaji T, Nunokawa M, Kubota H. Endovascular coil embolization for an anomalous splenic artery aneurysm with a splenomesenteric trunk. J Vasc Surg Cases Innov Tech. 2022;8:576–9. 10.1016/j.jvscit.2022.07.021. 36248400 10.1016/j.jvscit.2022.07.021 PMC9556584

[8] Kakamad FH, Hammood ZD, Salih AM, Abdalla BY, Mohammed KS, Karim SO, et al. Aneurysm of anomalous splenic artery arising from a splenomesenteric trunk: review of the literature with a report of a new case. Int J Surg Case Rep. 2021;80: 105618. 10.1016/j.ijscr.2021.02.004. 33592420 10.1016/j.ijscr.2021.02.004 PMC7893412

[9] Trastek VF, Pairolero PC, Joyce JW, Hollier LH, Bernatz PE. Splenic artery aneurysms. Surgery. 1982;91:694–9. 10.1007/BF01655271. 7079972

[10] Guillon R, Garcier JM, Abergel A, Mofid R, Garcia V, Chahid T, et al. Management of splenic artery aneurysms and false aneurysms with endovascular treatment in 12 patients. Cardiovasc Intervent Radiol. 2003;26:256–60. 10.1007/s00270-003-1948-y. 14562974 10.1007/s00270-003-1948-y

[11] Tulsyan N, Kashyap VS, Greenberg RK, Sarac TP, Clair DG, Pierce G, et al. The endovascular management of visceral artery aneurysms and pseudoaneurysms. J Vasc Surg. 2007. 10.1016/j.jvs.2006.10.049. 17264002 10.1016/j.jvs.2006.10.049

[12] Dong SL, Chen X, Tu ZX, Ai X, Zhang ZW, Guan Y, et al. Aneurysm of the anomalous splenic artery arising from superior mesenteric artery treated by coil embolization: a report of two cases and literature review. Ann Vasc Surg. 2018. 10.1016/j.avsg.2017.09.021. 29221838 10.1016/j.avsg.2017.09.021

[13] LaBella GD, Muck P, Kasper G, Welling R, Schlueter F, Vaughan A. Operative management of an aberrant splenic artery aneurysm: utility of the medial visceral rotation approach: a case report and review of the literature. Vasc Endovasc Surg. 2006;40:331–3. 10.1177/1538574406292005. 10.1177/1538574406292005 16959727

[14] De Cloedt L, Lavigne ChM, Lardinois F, Periquet Y, Verhelst R. Aneurysm of the splenic artery which arises from the superior mesenteric artery: a case report. Acta Chir Belg. 2010;110:332–4. 10.1080/00015458.2010.11680627. 20690517 10.1080/00015458.2010.11680627

[15] Feo CF, Scanu AM, Fancellu A, Costantino S. Visceral aneurysm and vascular anomaly involving the splenic artery. Dig Dis Sci. 2004;49:1378–80. 10.1023/b:ddas.0000042233.14587.ef. 15481306 10.1023/b:ddas.0000042233.14587.ef

[16] Hogendoorn W, Lavida A, Hunink MG, Moll FL, Geroulakos G, Muhs BE, et al. Open repair, endovascular repair, and conservative management of true splenic artery aneurysms. J Vasc Surg. 2014. 10.1016/j.jvs.2014.08.067. 25264364 10.1016/j.jvs.2014.08.067

[17] Sachdev U, Baril DT, Ellozy SH, Lookstein RA, Silverberg D, Jacobs TS, et al. Management of aneurysms involving branches of the celiac and superior mesenteric arteries: a comparison of surgical and endovascular therapy. J Vasc Surg. 2006;44:718–24. 10.1016/j.jvs.2006.06.027. 17011997 10.1016/j.jvs.2006.06.027

[18] Kulkarni CB, Moorthy S, Pullara SK, Kannan RR. Endovascular treatment of aneurysm of splenic artery arising from splenomesentric trunk using stent graft. Korean J Radiol. 2013;14:931–4. 10.3348/kjr.2013.14.6.931. 24265569 10.3348/kjr.2013.14.6.931 PMC3835641

[19] Kuwada N, Akagi D, Watanabe Y, Kanaoka Y, Tanemoto K. Endovascular stent graft placement and coil embolization for splenic artery aneurysm with an anatomical variant. Int J Angiol. 2023;32:273–6. 10.1055/s-0042-1742588. 37927835 10.1055/s-0042-1742588 PMC10624522

[20] Izumi H, Yoshii H, Fujino R, Takeo S, Nomura E, Mukai M, et al. Endovascular treatment of postoperative hemorrhage after pancreatectomy: a retrospective study. BMC Gastroenterol. 2023;23:379. 10.1186/s12876-023-03022-9. 37936060 10.1186/s12876-023-03022-9 PMC10631063

